# Elevated high-sensitivity cardiac troponin is associated with hypertrophy and fibrosis assessed with CMR in patients with hypertrophic cardiomyopathy

**DOI:** 10.1186/1532-429X-15-S1-P144

**Published:** 2013-01-30

**Authors:** Frank Gommans, Jeannette Bakker, Etienne Cramer, Michael A Fouraux, Maurice J Kurvers, Freek W Verheugt, Marc A Brouwer, Marcel Kofflard

**Affiliations:** 1 Cardiology, Radboud University Nijmegen Medical Centre, Nijmegen, Netherlands; 2 Cardiology, Albert Schweitzer Hospital, Dordrecht, Netherlands; 3 Radiology, Albert Schweitzer Hospital, Dordrecht, Netherlands; 4 Clinical Chemistry, Albert Schweitzer Hospital, Dordrecht, Netherlands

## Background

High-sensitivity (hs) cardiac troponin is a valuable biomarker of myocardial injury and frequently elevated in patients with hypertrophic cardiomyopathy (HCM). Using cardiovascular magnetic resonance (CMR) the HCM phenotype can be characterized in great detail with fibrosis as a key finding with prognostic impact. Therefore, our aim was to investigate whether elevated troponin levels in patients with clinical HCM were associated with LV hypertrophy and fibrosis as assessed with CMR.

## Methods

In 62 clinical HCM patients (58% males, mean age 51 ± 15 years) hs-troponin T was determined with a high-sensitivity assay (Roche Diagnostics). The lower detection limit is 0.003 pg/l and an elevated hs-troponin is defined as a concentration ≥ the 99^th^ percentile reference limit (≥ 0.014 pg/l). CMR with late gadolinium enhancement (LGE) was performed (Philips Achieva 1.5T) to assess LV mass indexed to body surface area, maximal wall thickness (MWT) and fibrosis. LV mass, MWT and the presence and extent of LGE were assessed (QMass 7.0, Medis) and compared between HCM patients with and without an elevated hs-troponin using Mann Whitney U or Fisher exact testing.

## Results

Hs-troponin was detectable in 46 HCM patients (74%), of whom 16 patients had an elevated hs-troponin level. The median LV mass and MWT were higher in HCM patients with an elevated hs-troponin (p<0.001). The difference in the presence of fibrosis was almost statistically significant between groups. Notably, if present, the extent of fibrosis was higher in patients with an elevated hs-troponin (p= 0.04) (Table 1).

**Table 1:** 

	Total N = 62	Troponin not elevated N = 46	Troponin elevated N = 16	P
LV mass indexed to BSA (g/m^2^) (median; IQR)	65; 52-91	61; 51-81	101; 67-130	0.001
Maximal LV wall thickness (mm) (median; IQR)	18; 13-21	16; 13-20	21; 18-24	0.002
Fibrosis present n (%)	31 (50%)	20 (44%)	11 (69%)	0.08
Fibrosis extent in LGE positive patients (%) (median; IQR)	10; 5-14	7; 3-12	14; 9-19	0.04

## Conclusions

Elevated troponin levels assessed with a high-sensitivity assay are common in HCM patients and associated with specific features of HCM such as hypertrophy and maximal wall thickness. Our findings are indicative of troponin as a potential surrogate marker of myocardial injury in the form of fibrosis. Future studies will have to address this issue more elaborately especially with regard to the potential prognostic value of troponin in these patients.

## Funding

None.

